# Familial Resemblance in Dietary Intakes of Children, Adolescents, and Parents: Does Dietary Quality Play a Role?

**DOI:** 10.3390/nu9080892

**Published:** 2017-08-17

**Authors:** Leonie H. Bogl, Karri Silventoinen, Antje Hebestreit, Timm Intemann, Garrath Williams, Nathalie Michels, Dénes Molnár, Angie S. Page, Valeria Pala, Stalo Papoutsou, Iris Pigeot, Lucia A. Reisch, Paola Russo, Toomas Veidebaum, Luis A. Moreno, Lauren Lissner, Jaakko Kaprio

**Affiliations:** 1 Department of Public Health, University of Helsinki, 00100 Helsinki, Finland; jaakko.kaprio@helsinki.fi; 2 Finnish Institute of Molecular Medicine, University of Helsinki, 00100 Helsinki, Finland; 3 Leibniz Institute for Prevention Research and Epidemiology—BIPS, 28359 Bremen, Germany; hebestr@leibniz-bips.de (A.H.); intemann@leibniz-bips.de (T.I.); pigeot@leibniz-bips.de (I.P.); 4 Population Research Unit, Department of Social Research, University of Helsinki, 00100 Helsinki, Finland; karri.silventoinen@helsinki.fi; 5 Department of Politics, Philosophy and Religion, Lancaster University, Lancaster LA1 4YL, UK; g.d.williams@lancaster.ac.uk; 6 Department of Public Health, Ghent University, 9000 Ghent, Belgium; Nathalie.Michels@UGent.be; 7 Department of Pediatrics, University of Pécs, 7622 Pécs, Hungary; molnar.denes@pte.hu; 8 Centre for Exercise, Nutrition and Health Sciences/School for Policy Studies, University of Bristol, Bristol BS8 1TH, UK; a.s.page@bris.ac.uk; 9 Department of Preventive and Predictive Medicine, Nutritional Epidemiology Unit, Fondazione IRCSS Istituto Nazionale dei Tumori, 20133 Milan, Italy; Valeria.Pala@istitutotumori.mi.it; 10 Research and Education Institute of Child Health, 2015 Strovolos, Cyprus; stalo.papoutsou@gmail.com; 11 Faculty 03: Mathematics and Computer Science, University of Bremen, 28359 Bremen, Germany; 12 Copenhagen Business School, Department of Management, Society and Communication, 2000 Frederiksberg, Denmark; lre.msc@cbs.dk; 13 Institute of Food Sciences, National Research Council, 83100 Avellino, Italy; prusso@isa.cnr.it; 14 Department of Chronic Diseases, National Institute for Health Development, 11619 Tallinn, Estonia; toomas.veidebaum@tai.ee; 15 GENUD (Growth, Exercise, Nutrition and Development) Research Group, Faculty of Health Sciences, University of Zaragoza, Instituto Agroalimentario de Aragón (IA2), Instituto de Investigación Sanitaria de Aragón (IIS Aragón) and Centro de Investigación Biomédica en Red de Fisiopatología de la Obesidad y Nutrición (CIBERObn), 50009 Zaragoza, Spain; lmoreno@unizar.es; 16 Section for Epidemiology and Social Medicine (EPSO), Institute of Medicine, Sahlgrenska Academy, University of Gothenburg, 405 30 Gothenburg, Sweden; lauren.lissner@gu.se

**Keywords:** familial aggregation, familial resemblance, familiality, shared environment, family study, dietary intake, diet quality, healthy diet, young children, adolescence

## Abstract

Information on familial resemblance is important for the design of effective family-based interventions. We aimed to quantify familial correlations and estimate the proportion of variation attributable to genetic and shared environmental effects (i.e., familiality) for dietary intake variables and determine whether they vary by generation, sex, dietary quality, or by the age of the children. The study sample consisted of 1435 families (1007 mothers, 438 fathers, 1035 daughters, and 1080 sons) from the multi-center I.Family study. Dietary intake was assessed in parents and their 2–19 years old children using repeated 24-h dietary recalls, from which the usual energy and food intakes were estimated with the U.S. National Cancer Institute Method. Food items were categorized as healthy or unhealthy based on their sugar, fat, and fiber content. Interclass and intraclass correlations were calculated for relative pairs. Familiality was estimated using variance component methods. Parent–offspring (*r* = 0.11–0.33), sibling (*r* = 0.21–0.43), and spouse (*r* = 0.15–0.33) correlations were modest. Parent–offspring correlations were stronger for the intake of healthy (*r* = 0.33) than unhealthy (*r* = 0.10) foods. Familiality estimates were 61% (95% CI: 54–68%) for the intake of fruit and vegetables and the sum of healthy foods and only 30% (95% CI: 23–38%) for the sum of unhealthy foods. Familial factors explained a larger proportion of the variance in healthy food intake (71%; 95% CI: 62–81%) in younger children below the age of 11 than in older children equal or above the age of 11 (48%; 95% CI: 38–58%). Factors shared by family members such as genetics and/or the shared home environment play a stronger role in shaping children’s intake of healthy foods than unhealthy foods. This suggests that family-based interventions are likely to have greater effects when targeting healthy food choices and families with younger children, and that other sorts of intervention are needed to address the intake of unhealthy foods by children.

## 1. Introduction

Parents provide both genes and the home environment for their children, and as such, are central in shaping children’s early experiences with food and eating behavior. Parents and other caregivers have considerable control over the foods that their children eat through the types of foods made available in and out of the home, food preparation methods, food-related parenting style, frequency of family meals, and deciding where the family goes out to eat [1,2]. For example, the availability of fruits, vegetables, and dairy foods in the household is an important predictor of children’s intake of these foods [2,3].

Children have a genetic predisposition to prefer foods that are sweet and salty and reject those that are sour and bitter, such as found in some vegetables [4]. They can, however, learn to accept these tastes through repeated exposure [5]. During family meals, parents or other familiar adults can serve as important role models for children’s willingness to try novel foods [6]. Eating meals together has been associated with healthful dietary patterns that track into adulthood, including increased intakes of fruits and vegetables, calcium-rich foods, fiber and micronutrients, and reduced intakes of fried food and soft drinks [7,8].

As children enter adolescence, they become more independent of their parents and have greater autonomy over their food intake. Peers exert an increasing influence on their snack and soft drink consumption, particularly when the availability of these foods at schools is high [9]. As children progress from childhood to adolescence, they are less likely to participate in family dinners at home [7], more likely to skip breakfast [10], and their diet quality and diet variety declines [10,11].

Previous research suggests that the parent–offspring resemblance in dietary intake is weak and that factors other than parental diet and home environment play an important role in influencing dietary intake in children and young adults [12,13,14]. As Wang et al. [13] previously summarized in a review of the literature and meta-analysis, most previous studies are based on small sample sizes and the resemblance varies by nutrients, foods and parent-child pairs. In a large study of 2692 child-parent pairs from the U.S., there were no consistent differences by age, i.e., the parent-child resemblance for the healthy eating index was stronger for children below the age of 10 year-old than older children, while the parent-child resemblance for energy, most nutrients and soft drinks was stronger for children older than 10 year-old than younger children [14].

In order to develop effective family-based interventions and target them appropriately, it is important to know whether familial or non-familial factors are more important in determining children’s food intake and whether the family environment exerts a stronger effect in younger than older children. For example, if familial effects weaken as children get older it may be advisable to introduce individual and peer related interventions instead of familial interventions to achieve dietary behavior change. In the I.Family study, the inclusion of a large number of families and children of a wide age range allowed us to calculate familial correlations and familiality estimates for usual intakes of energy, 4 macronutrients and 13 food groups and determine whether these correlations vary by generation, sex, types of foods or between younger and older children.

## 2. Materials and Methods

### 2.1. Sample

As part of the I.Family study, the six-year follow-up of the IDEFICS children (aged 2 to 9.9 years at baseline), their siblings and parents was conducted in 2013 [15]. The overriding aim is to understand how to prevent overweight and obesity in children and to identify determinants of eating habits, lifestyle choices, and health in European families. The families were recruited from eight European countries: Belgium, Cyprus, Estonia, Germany, Hungary, Italy, Spain and Sweden. From September 2007 to June 2008 (T_0_), 16,228 children (2 to 9.9 years old) were included in the baseline survey of the IDEFICS study [16]. Collection of nationally representative samples was not feasible, so two or more communities in each country whose socio-demographic profile and infrastructure were similar and typical for their region were selected. Two years after the baseline, all participants were invited for follow-up examinations (T_1_) where 11,041 (68%) participated. To take advantage of the setting-based recruitment, participation was also offered to all classmates of study participants who were not yet included at the baseline. Thus, 2555 children were newly recruited at T_1_. In 2012, the I.Family study commenced, with the aim to also enroll parents and siblings of children who had already participated in the IDEFICS study. In this way, 6167 families with an average of two children participated in the I.Family study. Ethics approval was obtained from responsible committees in each country. Parents and children older than 16 years provided written informed consent, while children aged 12 and over gave simplified written consent. Younger children gave oral consent for examinations and sample collection.

### 2.2. Interview on Kinship and Household

The parent or legal guardian took part in an interview on kinship and household composition. The interview was conducted using a Computer Assisted Telephone Interviewing (CATI), Computer Assisted Personal Interviewing (CAPI), face-to-face interview or pen-and-paper versions. The interview inquired about all adults and children living in the same household including each person’s relationship (biological or non-biological) to the child who already participated in the IDEFICS study. Family relationship codes were assigned for each person in the household. If a family had multiple children who already participated in the IDEFICS study, the interview inquired about each person’s relationship to the oldest participating child. The questions were repeated for all children and adults in the household.

### 2.3. Dietary Intake Assessment

Dietary intake was assessed using an online 24-h dietary recall (24HDR) assessment program, called “Self-Administered Children, Adolescents, and Adult Nutrition Assessment” (SACANA), based on the SACINA offline version [17]. This was based on a previously developed software used in adolescents, called the HELENA-Dietary Assessment Tool [18]. Children and parents were asked to recall their diet and to enter the type and the amount (g) of all foods and beverages consumed during the previous day, starting with the first intake after waking up in the morning. Parents were asked to proxy report the intake for younger children and/or assist children in filling in the 24HDR, especially those below the age of 11 years. Children aged 10 or younger were assisted by one of their parents more often than older children (77% vs. 55%). Standardized food images were used to assist portion size estimation. All nutrients and energy values were expressed per 100 g edible portion. The FFQ provided national examples of food groups common in the respective population and the SACANA food-composition tables provided all foods and beverages typically consumed in the populations, thus taking into account food intake by ethnic minorities. The participants were asked to complete repeated 24HDRs, including two working days and one weekend day, but the availability of repeated 24HDRs varied among individuals. Missing or implausible values for intakes of single food items that could not be corrected were imputed by country, food group, and age-specific median intakes (0.15% of the entries). Incomplete 24HDRs (recalls that have not been completed throughout) and those with more than four imputed values were excluded from the analysis. Age- and sex-specific Goldberg cut-offs were applied to classify each recall day as under-reported, plausibly reported, and over-reported energy intake [19].

After exclusion of under- and over-reporters, individual usual daily energy intake (energy intake in kcal/day), macronutrient intakes (g/day), and food group intakes (g/day) were estimated based on the U.S. National Cancer Institute Method [20,21]. This method allows the inclusion of covariates such as age and additional information from the food frequency questionnaire (FFQ), accounts for different intakes on weekend vs. working days and corrects for the variance inflation caused by the daily variation in dietary intake. Usual intakes were estimated for children as well as their parents on a sex-specific basis. Where available, multiple 24HDRs (one to four) per person were included in the calculation of usual daily intake (46% of the total sample had one recall day, 25% had two recall days, 26% had three recall days, and 3% had four recall days). Age was considered as a covariate in all models. When estimating usual food intakes, the corresponding food consumption frequency obtained from the FFQ was also used as a covariate to improve estimates (except for mixed dishes as this food group was not queried in the food frequency questionnaire but was a generic category in SACANA food groups). The I.Family FFQ consisted of 59 food items with possible answers ranging from “never/less than once a week”, “1–3 times a week”, “4–6 times a week”, “1 time/day”, “2 times a day”, “3 times a day”, and “I have no idea”. The FFQ allowed the categorization of food items as healthy or unhealthy, as mentioned above. Age-group specific residual variance parameters were used for five different age groups in children to allow the within-person variance to change by age.

Each food recorded by SACANA was assigned to one of these food categories: healthy cereals and cereal products (sugar < 15%, fat < 15% and fiber ≥ 5%), unhealthy cereals and cereal products (sugar ≥ 15%, fat ≥ 15% or fiber < 5%), unhealthy sugar and sweets (for example, candies, chocolate, nut spreads, jam, or ice cream), healthy fats & oils (from mainly plant origin and <40% fat for sauces), unhealthy fats & oils (mainly animal and processed origin and ≥40% fat for sauces), healthy fruits and vegetables (fresh fruits and vegetables, their fresh juices or lean preparation, without added sugars), healthy meat and meat products (containing < 10% fat, and meat products with <20% fat from poultry, rabbit or game), unhealthy meat and meat products (meats from all other origins than poultry, including offal, with ≥10% fat and meat products containing ≥20% fat), healthy meat alternatives (for example soy products, meat, and dairy substitutes), healthy milk and milk products (low fat and unsweetened), unhealthy milk and dairy products (full fat and sweetened, flavored), healthy mixed dishes (for example based on cereals, legumes, or vegetables), and unhealthy mixed dishes (for example fried foods, fast food, and snack foods) [22]. The healthy and unhealthy food groups were summed up to represent the sums of healthy and unhealthy foods.

Usual macronutrient intake was expressed as percentages of energy intake and food groups as grams per 1000 kcals.

### 2.4. Inclusion Criteria for the Final Study Sample

**Figure 1:**
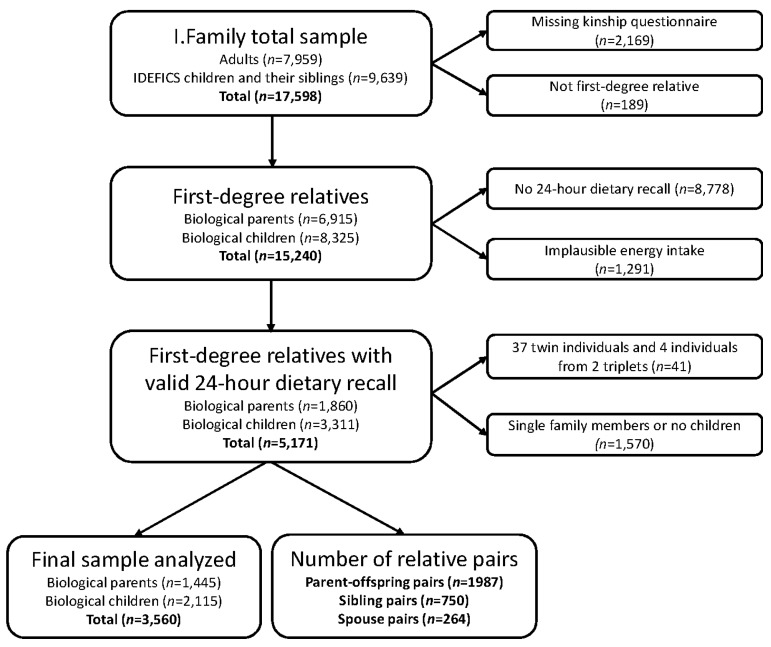
Flowchart showing the process leading to the final sample analyzed.

The flowchart showing the final sample analyzed is shown in Figure 1. From the total I.Family sample of 17,598 individuals, 15,429 provided information on the kinship interview, of whom 15,240 were first-degree relatives. Half-siblings were excluded because there were fewer than 20 in the sample. SACANA data were available for 6462 of these subjects of whom 1291 were excluded due to implausible energy intakes, the vast majority of which were under-reported (>97%). Because zygosity of twins and triplets was unknown, we randomly selected one individual of each twin pair or triplet to remain in the dataset (four individuals from two triplet sets and 37 individuals from 37 twin pairs were excluded). As we are assessing familial aggregation, families with only one eligible participant were excluded. Our eligible study population consisted of 1445 biological parents and 2115 children who belonged to 1435 nuclear families, of which 277 families were from Italy, 229 from Estonia, 137 from Cyprus, 67 from Belgium, 300 from Sweden, 262 from Germany, 107 from Hungary, and 56 from Spain. Thus all children included in this analysis were full siblings and biological offspring of their parents and were living in the same household (at least 50% of the time).

### 2.5. Statistical Analysis

Outlier identification and covariate adjustment were performed within each of the four sex-by-generation groups (mothers, fathers, daughters, and sons) and for the age-specific analysis for mother, father, younger daughters (2–10 year-old), older daughters (11–19 year-old), younger sons (2–10 year-old), and older sons (11–19 year-old) using Stata (version 13.0, STATA Corporation, College Station, College Station, TX, USA ). For each dietary variable, outliers were identified as being more than three standard deviations from the mean and were excluded. Skewed variables were log transformed before the regression analysis to achieve normally distributed residuals. Since the association between age and dietary intake may vary over the lifespan and may not be linear, stepwise multiple regression analysis was used for each variable up to a cubic polynomial in age (age, age^2^, age^3^), adjusted by country. Residuals from these regression models were then standardized to a mean of zero and standard deviation of one within each sex-by-generation group. Intra- and interclass correlations were calculated by using the FCOR program in the Statistical Analysis for Genetic Epidemiology software (SAGE, version 6.3, Case Western Reserve University, Cleveland, OH, USA) package [23]. FCOR calculates multivariate familial correlations with their asymptotic standard errors without assuming multivariate normality of the traits across family members [24]. It calculates familial correlations for all relative pair types available in the sample pedigrees. A homogeneity test implemented in FCOR was performed for the different parent–offspring and sibling correlations. This is a test of the hypothesis that all subtypes within a main type have the same correlation. The main types are grouped by non-sex specific relationship type. For example, the parent–offspring main type has four subtypes: father–son, father–daughter, mother–son, and mother–daughter. Familial correlations were computed for nine types of related pairs. Parent–offspring: mother–daughter, mother–son, father–daughter, father–son; siblings: sister–sister, brother–sister, and brother–brother; and for unrelated spouse pairs (mother–father). Furthermore, parent–offspring and sibling correlations were calculated for parent–younger children (2–10 year-old) and parent–older children (11–19 year-old), younger siblings vs. younger siblings (2–10 year-old) and older siblings (11–19 year-old) vs. older siblings (11–19 year-old) to investigate whether the resemblance differs for children versus adolescents.

The tests for the difference between two correlation coefficients were conducted following Cohen and Cohen (for independent correlation coefficients) [25]. If the correlation coefficients were derived from the same group, which was the case e.g., for parent–offspring, sibling, and spouse correlation coefficients of healthy and unhealthy foods, the test for dependent samples provided in the cocor R package was used instead [26,27].

Finally, we quantified familiality, that is the proportion of the observed phenotypic variance of a particular trait, in a particular population, which is attributable to all factors shared among family members using a maximum-likelihood variance component method implemented in SOLAR (Sequential Oligogenic Linkage Anaysis Routines) (Southwest Foundation for Biomedical Research). The variance decomposition method implemented in SOLAR is based on the fact that biologically related relatives share a certain amount of genes identity-by-descent (IBD) [28]. Thus the correlations between any pair of relatives depend on their degree of genetic and shared environmental relationships. Parent–offspring and sibling pairs are assumed to be correlated 0.5 for genetic factors when there is random mating for the trait being studied. All types of family members living in the same household are correlated unity (1) for the shared environmental factors. Unique environmental effects are those effects that make family members different from one another and are not correlated between them. This component also includes measurement error. We further investigated whether the familiality estimates vary between younger and older children. Because we only have nuclear families, genetic factors cannot be disentangled from familial environmental sources of resemblance. Therefore, our estimates of familiality should not be confused with heritability estimates from genome-wide association studies (GWAS) or twin and pedigree studies, which refer to the proportion of the total variance in a particular trait due to genetic factors (measured genes for GWAS or total inferred genes for twins or pedigrees) only [29]. All statistical tests are not adjusted for multiple testing. Thus, *p*-values should be interpreted with caution.

## 3. Results

### 3.1. Distribution of Family Types

Table 1 shows the distribution of family types where “family” refers to the members of the family participating in the present study. The most common family type was a mother with one child. The mean (±SD) family size in our study population was 2.5 (±0.7), ranging in size from two to six individuals, where all family members lived in the same household. The mean sibship size was 1.5 (±0.6) (ranging from zero to four). The mean and age ranges of parents and children were 43 years (25–65) and 11 years (2–19 years), respectively. The mean and age ranges of younger children were 9 years (2–10 year-old) and of older children 13 years (11–19 year-old), respectively. Less than 5% of the children were below the age of 7 years.

### 3.2. Characteristics of the Family Members

Table 2 shows the basic characteristics of the parents and their children. The mean and standard deviation for dietary variables are shown for mothers, fathers, younger daughters, younger sons, older daughters, and older sons separately. There were 1007 mothers, 438 fathers, 1035 daughters (of whom 517 younger), and 1080 sons (of whom 551 younger).

### 3.3. Familial Correlations

Parent–offspring and sibling correlations for dietary intake are shown in Table 3. The correlations showed familial resemblance for all dietary variables, except for the correlation of energy intake in older siblings. Correlations between parent and offspring ranged from *r* = 0.11–0.33 and between siblings from *r* = 0.21–0.43. Sibling correlations were significantly larger than parent–offspring correlations for the majority of traits. Spouse correlations were highest for protein, fruit and vegetables, healthy milk and dairy products, and healthy meat alternatives, and were weakest for unhealthy mixed dishes, unhealthy fat and oils, and unhealthy meat and meat products. Table 3 further shows the parent–offspring and sibling correlations for younger and older children separately. Parents were more similar to their younger children’s intake for fruit and vegetables compared to their older children’s intake. Younger siblings tended to be more alike in dietary intake than older siblings.

Table 4 shows the parent–offspring correlations for the four sex-by-generation groups and the sibling correlations for the same sex and opposite sex sibling pairs. Little heterogeneity in food intake was observed between the different dyads by sex. Heterogeneity by sex was only observed for healthy meat alternatives and unhealthy meat and meat products within the different parent–offspring pairs, and for healthy meat alternatives and healthy mixed dishes within sibling pairs.

### 3.4. Familiality Estimates

The proportions of the variance attributable to familial effects are presented in Table 5. The proportion of the phenotypic variance explained by familial factors was largest for fruit and vegetable intake. The remaining variance was attributable to unique environmental factors, that is, environmental factors that are not shared among family members. Table 5 further shows the familiality estimates for younger and older children separately. The familiality estimates for fruit and vegetables intake of the two age groups differed significantly (the 95% confidence intervals were not overlapping), i.e., familial factors contributed to a larger proportion of variance in the intake of fruit and vegetables for younger than older children.

**Figure 2:**
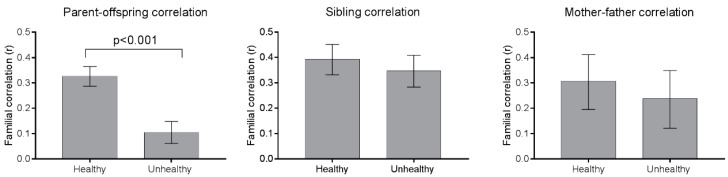
Parent–offspring, sibling correlations and spousal correlations for the sum of healthy and unhealthy food groups. Using Fisher’s *r*-to-*z* transformation, the confidence intervals are calculated using approximate standard errors [30]; the *p*-values are calculated according to [26]. The sample size was 1987 parent–offspring dyads, 750 sibling dyads, and 264 spouse pairs.

Healthy and unhealthy food groups were summed up to represent the total intake of healthy and unhealthy foods, and familial correlations for these sums are shown in Figure 2. Parent–offspring correlations were significantly stronger for healthy (*r* = 0.33) than unhealthy foods (*r* = 0.10) (*p <* 0.001 for the difference between the two correlation coefficients).

**Figure 3:**
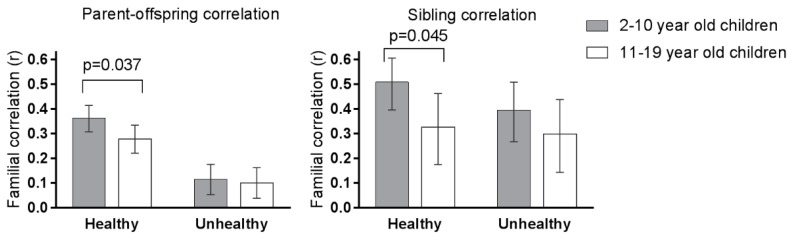
Parent–offspring and sibling correlations for the sum of healthy and unhealthy food groups separately for younger and older children. Using Fisher’s *r*-to-*z* transformation, the confidence intervals are calculated using approximate standard errors [30]; the *p*-values are calculated according to [25]. The sample size was 996 Parent–offspring dyads for the younger children, 991 Parent–offspring dyads for the older children, 192 younger sibling dyads, and 149 older sibling pairs.

Parent–offspring and sibling correlations for healthy and unhealthy foods are shown separately for younger and older children in Figure 3. For the intake of healthy foods, parents’ resemblance to their children differed significantly between younger and older children (*r* = 0.36 for younger and *r* = 0.28 for older children, *p* = 0.04 for the difference between the two correlation coefficients). Parent–child resemblance in the intake of unhealthy foods did not vary by the age of the children and was low for both younger (*r* = 0.12) and older (*r* = 0.10) children. Sibling correlations for the intake of healthy foods were stronger for younger than older siblings (*r* = 0.51 for younger and *r* = 0.33 for older siblings, *p* = 0.045 for the difference between the two correlation coefficients). Sibling resemblance in the intake of unhealthy foods did not differ significantly between younger (*r* = 0.40) and older (*r* = 0.30) siblings.

**Figure 4:**
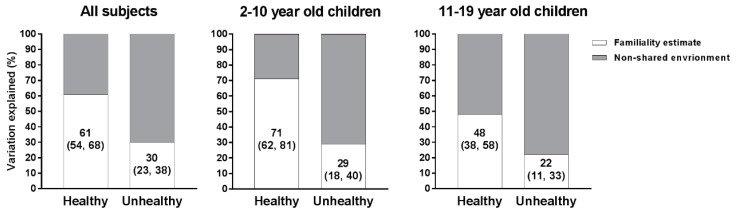
Porportion of variation for the sum of healthy and unhealthy food groups explained by familial and non-familial factors for all subjects and separately for younger and older children. The sample size consisted of 1435 families (746 families in the younger age group, and 760 families in the older age group).

The familiality estimates for the sums of healthy and unhealthy food groups are shown in Figure 4. Familial factors explained 61% of the variation in the intake of healthy foods, and only half as much (30%) of the variation in the intake of unhealthy foods. For the intake of unhealthy foods, familiality estimates were low for both younger and older children (29% and 22%, respectively), while the familiality estimate for the sum of healthy foods dropped from 71% in younger children to 48% in older children.

## 4. Discussion

The results of this large multi-center European study confirm a substantial familial resemblance in usual intake of energy, macronutrients, and food groups. These may arise from shared genetic factors between biologically-related relatives, shared family environmental factors between family members living in the same household, and assortative mating or social homogamy between spouses. Our parent–child correlations are consistent with the magnitude of correlations reported by Wang et al. in their review and meta-analysis on parent-child resemblance in dietary intake [13]. The current study extends this knowledge, by showing firstly, that familial correlations—particularly parent–offspring correlations—are stronger for healthy food intake than unhealthy food intake; and secondly, that familial resemblance, particularly parent–offspring correlations is stronger for younger children aged 2–10 than for older children aged 11–19.

In the present study, familial factors explained 61% of the variance in the intake of healthy foods and only half as much (30%) in the intake of unhealthy foods. A possible explanation could be that children have a strong biological preference for sweet and salty foods and this preference decreases with increasing age and as they enter adulthood [31,32]. Thus, the age difference between parents and children could contribute to the weak parent–child resemblance in unhealthy food intake in the present study. Another explanation could be that in the European societies in which the present study was conducted, there are more external influences on unhealthy food intake than healthy food intake. For example, television viewing has been shown to influence obesity risk in children and to contribute to overconsumption of higher-fat and higher-sugar diets [33]. Since advertising is often targeted specifically at children, children can be assumed to be affected by advertising from an early age onwards. Most foods marketed to children on television are for products high in sugar, fat, and sodium such as fast food, sweets, snacks, and unhealthy cereals [34]. Food advertising could lead to the child requesting the food through what has been termed the “nag factor” or “pester power” and could consequently influence what children eat [35]. This could further contribute to parent–child dissimilarity in the intake of foods high in sugar and fat. Friends and peers could also contribute to the lower resemblance between parents and their children in unhealthy food intake. Among students, it has been shown that attitudes towards fruit were predicted by those of their parents rather than friends, while preference for sweet was predicted by friends’ rather than parents’ attitudes, suggesting parental influence as regards healthy foods and peer influence as regards junk food [36]. This could be mediated by social norms, as it has been reported that adolescents tend to associate healthy food with family and junk food with friends, and these associations predict food consumption frequency [37].

We further found that parents resemble their younger children’s intake of healthy foods more than their older children’s intake of the same foods, and familial factors are a more important factor in determining variation in healthy food intake among younger than older children. In contrast, for the intake of unhealthy foods, no differences were seen between younger and older children. As younger children consume most of their main meals at home, the home environment is likely to be the main determinant of the intake of healthy foods, including fruit and vegetables. If such foods are not made available to children at home, children are unlikely to consume them outside the home. Family meals have been found to be associated with more healthful eating patterns, and the frequency of family meals declines as children become older and more independent [7]. Longitudinal research has shown that as children move from elementary to junior high and middle school, their consumption of breakfast, fruits, vegetables, and milk decreases and the popularity of soft drink consumption increases [10]. One previous study reported the parent–child correlations for the healthy eating index to be stronger for children below the age of 10 than for children above the age of 10, whereas for fiber, calcium and some other nutrients, the resemblance was stronger for older than younger children [14]. Direct comparison with the current study is difficult as only very few food groups were examined in that study.

Overall, we found few sex differences. For a few nutrients and food groups, including unhealthy milk and dairy products and unhealthy meat and meat products, father–offspring dyads were not correlated. Furthermore, for unhealthy meat and meat products and healthy meat alternatives, heterogeneity was found for the different subtypes, and mother–child correlations tended to be stronger than father–child correlations. Some previous studies have reported stronger correlations between mothers and daughters in dietary intake than between mothers and sons [38,39], whereas others did not detect any sex differences [40,41]. In a population-based sample of 471 mother–child pairs from Stockholm, there were significant associations between mothers and sons in the intake of many food groups. Overall, however, mothers and daughters were more strongly correlated in their dietary intakes than mothers and sons, and macronutrient and energy intakes were significantly correlated between mothers and daughters, but not between mothers and sons [39]. Mothers usually devote more time to child care than fathers, whether they are employed or not [42], and women are more likely to take primary responsibility in meal planning, shopping, and preparation than men [43,44]. In addition, long working hours and schedules among fathers were found to be positively associated with take-out meals, missed family meals, prepared entrees, and eating while working [45]. Thus the greater resemblance of mother–child than father–child pairs in some aspects of dietary intake could be explained by the additional environmental factors that mothers share with their children as compared to fathers, such as the time that mothers devote to food preparation and feeding their children. Furthermore, stronger maternal associations could also suggest maternal in utero programming of offspring appetite by maternal intake during pregnancy [46]. It is unlikely that non-paternity, which is estimated to be only about 1–3% for Western populations [47,48] has significantly contributed to the few correlations that are lower between children and their fathers versus their mothers.

When we compare our sibling correlations to those reported in other studies, we find larger correlations than previously reported. For example, in a prospective cohort of postmenopausal women from the Iowa Women’s Health Study, sibling correlations for nutrient intakes ranged from 0.04 to 0.17 [49], while sibling correlations for dietary traits in the present analysis ranged from 0.21 to 0.43. It is likely that this difference is due to the fact that siblings in the I.Family study still live in the same home, whereas the adult siblings in other studies generally no longer share these home environmental factors. We found that correlations were about the same for sister–sister, sister–brother, and brother–brother pairs, and little evidence that siblings of the same sex would share a more common social environment than siblings of the opposite sex.

Mothers and fathers were modestly correlated for all dietary factors examined, and for many dietary factors, these were of the same magnitude as parent–offspring correlations, suggesting a shared household effect. Alternatively, assortative mating can contribute to spouses’ resemblance, and this has been well documented for height [50], obesity [50,51], and lifestyle behaviors [52,53]. Interestingly, the strength of the spousal correlation did not differ between healthy and unhealthy food groups, suggesting that home environment influences the intake of healthy and unhealthy food groups to an equal extent in adults.

The present analysis has important strengths, including the recently collected large sample of families with information on biological relatedness and in-depth phenotyping of children and their parents which allowed a comprehensive assessment of habitual dietary intake. In order to increase the accuracy of the dietary assessment, we used a computer-assisted 24HDR that included standardized photographs, multiple plausibility checks, and reminding questions that facilitated reporting of accurate portion sizes and complete recalls. We corrected for reporting bias by excluding incomplete recalls and recalls with implausible energy intakes.

However, our study also has limitations that should be considered when interpreting our findings. We acknowledge that the estimate of familiality in this study is not directly comparable to the heritability estimates obtained from previous twin and pedigree studies. Unfortunately, we cannot distinguish between genetic and shared environmental effects. Many twin studies also report an estimate for shared environment effects together with heritability this can be cautiously compared to familiality estimates. Our study of nuclear families does not allow us to conclude whether the familiality estimate is due to genetic or shared environmental factors, and whether the lower familiality estimate in older children is due to stronger genetic influences in younger children or diminished shared environmental influences in older children. Previous twin studies suggest that genetic influences are largest and shared environmental influences lowest for healthy foods including fruits, vegetables, and protein [54,55]. This has been shown to vary by age since in young children shared environmental factors have a substantially stronger influence on food preferences than genetic factors, while in adolescence the shared environmental influence appears to disappear completely [55]. Although our overall sample size was large, it was lower when comparing the correlations between the different dyads by sex or age.

Children may have consumed some foods outside the home and this might have decreased the accuracy of 24HDR in younger children when proxy reports by parents were used. In addition, younger children more often required assistance in completing one or more 24HDR and this may have contributed to stronger similarity between parents and their younger children. However, less than 5% of participating children were below seven years of age. Previous research has shown that by the age of 8–10 years children can reliably report their food intake [56,57] and that children as young as 6 years can accurately recall their school lunch intake for one occasion while teachers record with less accuracy [58].

## 5. Conclusions

In conclusion, dietary intake significantly aggregates within European families, both among biologically related relatives, including parents and their children and siblings, and non-biologically related spouse pairs living in the same household. Factors shared by family members (such as genetics and the shared home environment) play a stronger role in shaping children’s intake of healthy foods than unhealthy foods, especially those of younger children. This suggests that family-based interventions are likely to have greater effects when targeting healthy food choices and families with younger children, and that other sorts of intervention are needed to address the intake of unhealthy foods by children.

## Figures and Tables

**Table 1:** Distribution of family types in the study population.

Family Types	Number of Families	Percentage	Number of Individuals
Mother, 1 child	536	37.4	1072
Mother, 2 children	195	13.6	585
Mother, 3 children	10	0.7	40
Mother, 4 children	2	0.1	10
Father, 1 child	129	9.0	258
Father, 2 children	38	2.7	114
Father, 3 children	6	0.4	24
Father, 4 children	1	0.1	5
Mother, father, 1 child	154	10.7	462
Mother, father, 2 children	89	6.2	356
Mother, father, 3 children	18	1.3	90
Mother, father, 4 children	3	0.2	18
2 children	236	16.45	472
3 children	18	1.15	54
Total	1435	100	3560

**Table 2:** Dietary intakes of mothers, fathers, daughters, and sons

	Mothers	Fathers	Daughters 2–10 Year-Old	Sons 2–10 Year-Old	Daughters 11–19 Year-Old	Sons 11–19 Year-Old
*N*, number of subjects	1007	438	517	551	518	529
Total energy (kcal)	1707 ± 154	2254 ± 192	1605 ± 131	1760 ± 182	1809 ± 141	2082 ± 204
Macronutrients (% energy intake)						
Total fat	37 ± 4	36 ± 4	33 ± 3	33 ± 3	34 ± 3	33 ± 3
Total carbohydrates	45 ± 5	44 ± 5	52 ± 4	52 ± 4	51 ± 4	51 ± 4
Total protein	16 ± 2	16 ± 1	15 ± 1	15 ± 1	15 ± 1	15 ± 1
Total sugar	19 ± 5	17 ± 4	21 ± 5	21 ± 4	20 ± 4	20 ± 3
Healthy food groups (g/1000 kcal)						
Healthy cereals and cereal products	35 ± 20	35 ± 22	22 ± 14	25 ± 13	21 ± 14	20 ± 10
Healthy fat and oils	10 ± 4	10 ± 4	11 ± 4	10 ± 3	10 ± 3	10 ± 3
Fruits and vegetables	155 ± 63	113 ± 47	97 ± 35	88 ± 31	89 ± 31	70 ± 27
Healthy meat and meat products	27 ± 5	26 ± 6	24 ± 5	23 ± 5	22 ± 5	24 ± 6
Healthy milk and dairy products	57 ± 42	50 ± 36	74 ± 60	81 ± 59	65 ± 52	64 ± 48
Healthy meat alternatives	23 ± 13	22 ± 11	15 ± 7	16 ± 10	12 ± 6	14 ± 10
Healthy mixed dishes,	57 ± 14	55 ± 8	46 ± 9	43 ± 11	51 ± 8	47 ± 10
Unhealthy food groups (g/1000 kcal)						
Unhealthy cereals and cereal products	48 ± 14	55 ± 21	77 ± 18	76 ± 20	70 ± 16	78 ± 19
Unhealthy fat and oils	11 ± 5	10 ± 5	9 ± 3	11 ± 4	10 ± 3	10 ± 3
Sugar and sweets	46 ± 22	37 ± 19	49 ± 22	47 ± 12	47 ± 22	41 ± 11
Unhealthy meat and meat products	23 ± 5	31 ± 4	29 ± 5	28 ± 4	27 ± 5	27 ± 4
Unhealthy milk and dairy products	56 ± 42	46 ± 39	68 ± 41	70 ± 50	56 ± 32	57 ± 43
Unhealthy mixed dishes	56 ± 8	53 ± 8	53 ± 6	54 ± 7	60 ± 6	60 ± 7
Sum of healthy and unhealthy food groups (g/1000 kcal)						
Healthy foods	364 ± 89	311 ± 67	289 ± 80	285 ± 76	271 ± 69	249 ± 64
Unhealthy foods	240 ± 47	231 ± 45	285 ± 46	285 ± 50	271 ± 39	273 ± 45

Data are means ± SD.

**Table 3:** Parent–offspring and sibling correlations in dietary intake for all subjects and separately for younger and older children.

	Parent–Offspring (*n* = 1987)	Sibling-Sibling (*n* = 750)		Parent–Offspring 2–10 Year-Old (*n* = 996)	Parent–Offspring 11–19 Year-Old (*n* = 991)		Sibling-Sibling 2–10 Year-Old (*n* = 192)	Sibling-Sibling 11–19 Year-Old (*n* = 149)		Mother-Father (*n* = 264)
	*r*	*r*	*p*-Value ^a^	*r*	*r*	*p*-Value ^a^	*r*	*r*	*p*-Value ^a^	*r*
Total energy (kcal)	0.16	0.28	0.004	0.13	0.17	0.38	0.38	0.14	0.02	0.23
Macronutrients (% energy intake)										
Total fat	0.21	0.32	0.01	0.21	0.22	0.92	0.31	0.26	0.63	0.25
Total carbohydrates	0.21	0.31	0.02	0.18	0.24	0.19	0.33	0.32	0.90	0.26
Total protein	0.26	0.31	0.23	0.25	0.26	0.81	0.31	0.22	0.37	0.33
Total sugar	0.21	0.34	<0.001	0.22	0.18	0.38	0.37	0.17	0.05	0.26
Healthy food groups (gram/1000 kcal)										
Healthy cereals and cereal products	0.33	0.38	0.18	0.36	0.29	0.11	0.48	0.36	0.18	0.27
Healthy fat and oils	0.27	0.34	0.09	0.31	0.24	0.06	0.45	0.31	0.14	0.23
Fruits and vegetables	0.33	0.35	0.59	0.38	0.28	0.01	0.43	0.19	0.02	0.29
Healthy meat and meat products	0.21	0.40	<0.001	0.23	0.18	0.20	0.31	0.24	0.49	0.27
Healthy milk and dairy products	0.25	0.36	0.004	0.28	0.23	0.26	0.52	0.35	0.06	0.29
Healthy meat alternatives	0.23	0.21	0.67	0.26	0.21	0.19	0.39	0.30	0.41	0.29
Healthy mixed dishes	0.23	0.43	<0.001	0.25	0.19	0.18	0.52	0.30	0.02	0.22
Unhealthy food groups (gram/1000 kcal)										
Unhealthy cereals and cereal products	0.23	0.33	0.02	0.22	0.22	0.86	0.45	0.28	0.07	0.27
Unhealthy fat and oils	0.22	0.39	<0.001	0.25	0.19	0.24	0.50	0.27	0.02	0.17
Sugar and sweets	0.19	0.31	0.003	0.22	0.19	0.37	0.40	0.26	0.17	0.24
Unhealthy meat and meat products	0.20	0.37	<0.001	0.20	0.21	0.77	0.43	0.35	0.43	0.17
Unhealthy milk and dairy products	0.11	0.39	<0.001	0.12	0.10	0.63	0.42	0.30	0.25	0.21
Unhealthy mixed dishes	0.20	0.34	<0.001	0.19	0.19	0.84	0.46	0.20	0.01	0.15

^a^ The *p*-values for the difference between two familial correlation coefficients (Parent–offspring vs. sibling-sibling; parent-younger offspring vs. parent older-offspring; younger-siblings vs. older siblings) calculated according to [25]. All single familial correlations are statistically significant at α = 0.05 (*p*-values not shown), except for the correlation of energy intake in siblings 11–19 year-old (*p* = 0.09). The number of relative pairs may be lower for some dietary variables due to exclusion of outliers.

**Table 4:** Parent–offspring and sibling correlations (*r*) by sex dyads.

	Parent–Offspring					Siblings			
	Father–Son (*n* = 326)	Mother–Son (*n* = 705)	Father–Daughter (*n* = 299)	Mother–Daughter (*n* = 657)		Brother–Brother (*n* = 194)	Sister–Brother (*n* = 358)	Sister–Sister (*n* = 198)	
	*r*	*r*	*R*	*r*	*p*-Value ^a^	*r*	*r*	*r*	*p*-Value ^a^
Total energy (kcal)	0.18	0.14	0.16	0.17	0.95	0.40	0.20	0.30	0.05
Macronutrients (% energy intake)									
Total fat	0.16	0.20	0.27	0.25	0.39	0.33	0.31	0.32	0.96
Total carbohydrates	0.14	0.22	0.20	0.25	0.41	0.31	0.33	0.32	0.98
Total protein	0.23	0.26	0.28	0.25	0.93	0.38	0.24	0.32	0.26
Total sugar	0.09	0.22	0.25	0.22	0.43	0.39	0.29	0.35	0.42
Healthy food groups (gram/1000 kcal)									
Healthy cereals and cereal products	0.28	0.35	0.30	0.33	0.66	0.43	0.33	0.41	0.38
Healthy fat and oils	0.27	0.30	0.14	0.31	0.10	0.33	0.34	0.31	0.91
Fruits and vegetables	0.31	0.30	0.38	0.35	0.57	0.35	0.32	0.40	0.57
Healthy meat and meat products	0.32	0.24	0.17	0.21	0.25	0.30	0.20	0.11 ^b^	0.18
Healthy milk and dairy products	0.21	0.23	0.18	0.20	0.88	0.42	0.37	0.38	0.40
Healthy meat alternatives	0.21	0.32	0.19	0.22	0.004	0.53	0.37	0.22	0.002
Healthy mixed dishes	0.15	0.23	0.13	0.26	0.08	0.51	0.26	0.49	0.005
Unhealthy food groups (gram/1000 kcal)									
Unhealthy cereals and cereal products	0.28	0.24	0.25	0.23	0.99	0.31	0.32	0.38	0.38
Unhealthy fat and oils	0.21	0.27	0.15	0.21	0.37	0.49	0.34	0.38	0.11
Sugar and sweets	0.21	0.21	0.16	0.18	0.87	0.36	0.22	0.39	0.16
Unhealthy meat and meat products	0.06 ^b^	0.24	0.03 ^b^	0.25	<0.001	0.46	0.37	0.33	0.39
Unhealthy milk and dairy products	0.06 ^b^	0.11	0.06 ^b^	0.15	0.51	0.37	0.33	0.38	0.92
Unhealthy mixed dishes	0.15	0.16	0.13	0.30	0.02	0.44	0.30	0.31	0.17

^a^ The *p*-value for homogeneity among subtypes within each main type. ^b^ The familial correlation is not significant (*p* > 0.05). The number of relative pairs may be lower for some dietary variables due to exclusion if outliers. The number of relative pairs may be lower for some dietary variables due to exclusion of outliers.

**Table 5:** Familiality estimates for all subjects and for younger and older children.

	All Subjects (*n* = 3560)	Children 2–10 Year-Old (*n* = 1777)	Children 11–19 Year-Old (*n* = 1773)
	Familiality (95% CI) ^a^	Familiality (95% CI)	Familiality (95% CI)
Total energy (kcal)	0.35 (0.27, 0.42)	0.32 (0.22, 0.43)	0.29 (0.18, 0.40)
Macronutrients (% energy intake)			
Total fat	0.46 (0.39, 0.53)	0.43 (0.32, 0.54)	0.43 (0.32, 0.54)
Total carbohydrates	0.44 (0.37, 0.51)	0.39 (0.28, 0.50)	0.44 (0.33, 0.54)
Total protein	0.47 (0.40, 0.54)	0.48 (0.38, 0.58)	0.44 (0.34, 0.55)
Total sugar	0.44 (0.36, 0.51)	0.44 (0.34, 0.55)	0.33 (0.22, 0.45)
Healthy food groups (gram/1000 kcal)			
Healthy cereals and cereal products	0.59 (0.53, 0.66)	0.64 (0.55, 0.73)	0.53 (0.43, 0.63)
Healthy fat and oils	0.52 (0.45, 0.59)	0.61 (0.51, 0.71)	0.43 (0.32, 0.53)
Fruits and vegetables	0.61 (0.54, 0.68)	0.72 (0.62, 0.81)	0.50 (0.39, 0.60)
Healthy meat and meat products	0.41 (0.33, 0.48)	0.46 (0.36, 0.57)	0.38 (0.27, 0.49)
Healthy milk and dairy products	0.45 (0.38, 0.52)	0.49 (0.39, 0.60)	0.34 (0.24, 0.44)
Healthy meat alternatives	0.42 (0.35, 0.49)	0.52 (0.42, 0.62)	0.43 (0.32, 0.54)
Healthy mixed dishes	0.48 (0.41, 0.55)	0.49 (0.39, 0.59)	0.38 (0.27, 0.50)
Unhealthy food groups (gram/1000 kcal)			
Unhealthy cereals and cereal products	0.46 (0.38, 0.53)	0.43 (0.33, 0.53)	0.41 (0.30, 0.52)
Unhealthy fat and oils	0.46 (0.39, 0.53)	0.50 (0.40, 0.60)	0.37 (0.27, 0.48)
Sugar and sweets	0.42 (0.34, 0.49)	0.50 (0.39, 0.60)	0.34 (0.23, 0.45)
Unhealthy meat and meat products	0.44 (0.37, 0.51)	0.41 (0.31, 0.52)	0.41 (0.30, 0.52)
Unhealthy milk and dairy products	0.31 (0.24, 0.38)	0.29 (0.18, 0.40)	0.21 (0.10, 0.32)
Unhealthy mixed dishes	0.42 (0.35, 0.49)	0.39 (0.29, 0.49)	0.38 (0.27, 0.50)

The number of relative pairs may be lower for some dietary variables due to exclusion of outliers. ^a^ 95% CI = 95% confidence interval.Sum of healthy and unhealthy food groups.
